# Dendrimers as Nanocarriers for Nucleic Acid and Drug Delivery in Cancer Therapy

**DOI:** 10.3390/molecules22091401

**Published:** 2017-08-23

**Authors:** Livia Palmerston Mendes, Jiayi Pan, Vladimir P. Torchilin

**Affiliations:** 1 Center for Pharmaceutical Biotechnology and Nanomedicine, Northeastern University, Boston, MA 02115, USA; mendes.l@husky.neu.edu (L.P.M.); pan.jiay@husky.neu.edu (J.P.); 2 CAPES Foundation, Ministry of Education of Brazil, Brasilia 70040-020, Brazil

**Keywords:** dendrimers, PAMAM, PPI, PLL, cancer, nucleic acid, drug

## Abstract

Dendrimers are highly branched polymers with easily modifiable surfaces. This makes them promising structures for functionalization and also for conjugation with drugs and DNA/RNA. Their architecture, which can be controlled by different synthesis processes, allows the control of characteristics such as shape, size, charge, and solubility. Dendrimers have the ability to increase the solubility and bioavailability of hydrophobic drugs. The drugs can be entrapped in the intramolecular cavity of the dendrimers or conjugated to their functional groups at their surface. Nucleic acids usually form complexes with the positively charged surface of most cationic dendrimers and this approach has been extensively employed. The presence of functional groups in the dendrimer’s exterior also permits the addition of other moieties that can actively target certain diseases and improve delivery, for instance, with folate and antibodies, now widely used as tumor targeting strategies. Dendrimers have been investigated extensively in the medical field, and cancer treatment is one of the greatest areas where they have been most used. This review will consider the main types of dendrimer currently being explored and how they can be utilized as drug and gene carriers and functionalized to improve the delivery of cancer therapy.

## 1. Introduction

Dendrimers are highly branched polymeric macromolecules with well-defined and uniform sizes and shapes. Their basic structure comprises three main components: a central core, repetitive branching units, and terminal groups, that provide modifiable surface functionalities. The increase in the number of repeated branching units determines the generation of the dendrimer and is responsible for the formation of a globular structure [1,2,3]. The high level of control possible over their architecture makes them attractive as systems for drug [4,5] and gene [6,7,8] delivery applications. Drugs and oligonucleotides can be either encapsulated in their internal cavities or bound to their surfaces through hydrophobic or electrostatic interactions. They can also be covalently attached through reactions with the terminal functional groups. 

Since they were first reported in 1978 [9], dendrimers have been synthesized by two major routes: the divergent method, introduced by Tomalia [10], and convergent growth, developed by Hawker and Frechet [11]. There are, however, other less well-explored strategies to synthesize dendrimers, including hypercore and branched monomers growth [12], double exponential growth [13], lego chemistry [14], and click chemistry [15].

### 1.1. Dendrimer Synthesis: Divergent and Convergent Approaches

In the divergent growth method, the final molecule grows radially from a core by the sequential addition of layers of monomers, each layer constituting a new generation. The number of surface groups multiplies according to the functionalities in each monomer ramification [16,17]. It is important that every step of the reaction is fully completed before the addition of a new generation to avoid defects in the branches. One of the advantages of this approach is that in the final step of the synthesis reaction, the surface of the dendrimer can be easily modified with desired functional groups. It is also a reasonably fast synthesis which allows the preparation of large dendrimers. Among the downsides of this approach is the lengthy purification needed, since the final product and the intermediate reactants have similar molecular weights, charge, and polarity [16,18]. Also, by this method, the higher the generation, the greater the chances are of having branching defects, since the presence of bulky branches creates difficulties in the coupling of new ones [19]. Despite these obstacles, the advantages of this strategy have made it the most utilized route for dendrimer production to date.

In an opposite way from the divergent synthesis, dendrimers can also be synthesized starting from the surface using a convergent approach. The growth of the molecule starts from the ends of the chain, beginning by integrating the various branching points with other monomers that will constitute the dendrimers. Finally, these branches are attached to a central core when they reach the desired generation size [17,20]. In contrast to the divergent growth, this method permits easier purification due to bigger differences between the final products and the initial reagents. Other advantages include higher monodispersity for low generations and fewer branch defects. The main drawbacks are lower yield and difficulties in obtaining higher generations due to steric hindrances encountered when the branches are connected to the core [18].

### 1.2. Types of Dendrimers

**Figure 1:**
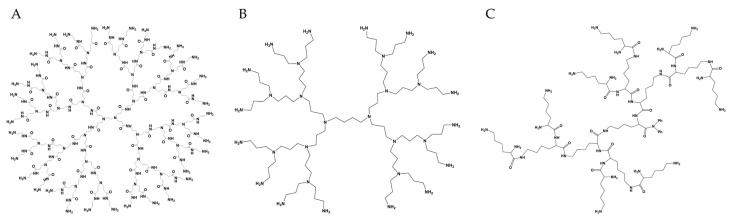
Schematic representation of third generation dendrimers. (**A**) PAMAM with ethylenediamine core; (**B**) PPI with 1,4-diaminobutane core; (**C**) PLL with Boc-l-Lys(Boc)-OH benzhydrylamide core. Illustrations generated by ChemDraw Professional Version 16.0.1.4.

A variety of dendrimers have been developed and used since the 1980s, but the ones derived from polyamidoamine (PAMAM) are undeniably the most employed (Figure 1A). They are hydrophilic, biocompatible, and non-immunogenic systems, which favors their use in drug delivery. The core of PAMAM is most commonly ethylenediamine [21], although more hydrophobic molecules—including diaminododecane, diaminoexane, and diaminobutane—can also be used [22,23]. Their branching units are based on methyl acrylate and ethylenediamine, and they have amine (in full generations) and carboxyl (in half generations) terminated groups [24].

Poly(propylene imine) (PPI) dendrimers (Figure 1B) were the first ones to be reported by Buhleier et al. in 1978 [9]. They referred to them as a cascade of molecules. Along with PAMAM, they were also widely studied. PPI dendrimers can be based on a 1,4-diaminobutane (DAB) core, but can also be synthesized from an ethylenediamine nucleus and other core molecules by a double Michael addition reaction. Propylene imine monomers are used as branching units. Hence their interior contains various tertiary tris-propylene amines, and they form full generations with primary amines as surface ends [25,26]. The presence of alkyl chains in their branching units imparts them a more hydrophobic interior than PAMAM dendrimers (which contain amide groups in addition to the alkyl chains) of equivalent generations [27,28].

Poly-l-lysine (PLL) dendrimers (Figure 1C) are a type of peptide dendrimer used mostly as gene carriers due to their excellent condensation with oligonucleotides [29]. Among their favorable characteristics are good biocompatibility, water solubility, biodegradability, and flexibility, similar to other dendrimers. With peptide bonds in their structures, both their core and branching units are commonly based on the amino acid lysine [30,31]. PLL dendrimers differ from the general concept of PAMAM and PPI dendrimers since they are mostly asymmetrical. However, they are still precise molecules, with a controlled number of lysines branching out from the core, and terminal amine residues [31,32]. The lysine in the terminal group of PLL contains two primary amines that are frequently modified for better biological performance [33,34].

### 1.3. Toxicity of Dendrimers

Despite showing great potential for biological applications, especially as drug and gene delivery agents, all classes of dendrimers present cytotoxic and hemolytic properties, which raises concern regarding their safety. The toxicity is dependent on the dendrimer characteristics and can be related to the chemistry of the core, but mainly to the surface end groups [35]. In most cases, these problems are related to the strong cationic characteristics of these nanoparticles [35,36,37]. As well as other positively charged polymers, the high charge and strong interaction with the negatively charged cell membranes can cause cell destabilization, with leakage of cytoplasmic proteins and subsequent lysis [37,38]. Thus, many recent studies involving dendrimers employ modification strategies to reduce or mask their charge and overcome these drawbacks.

**Figure 2:**
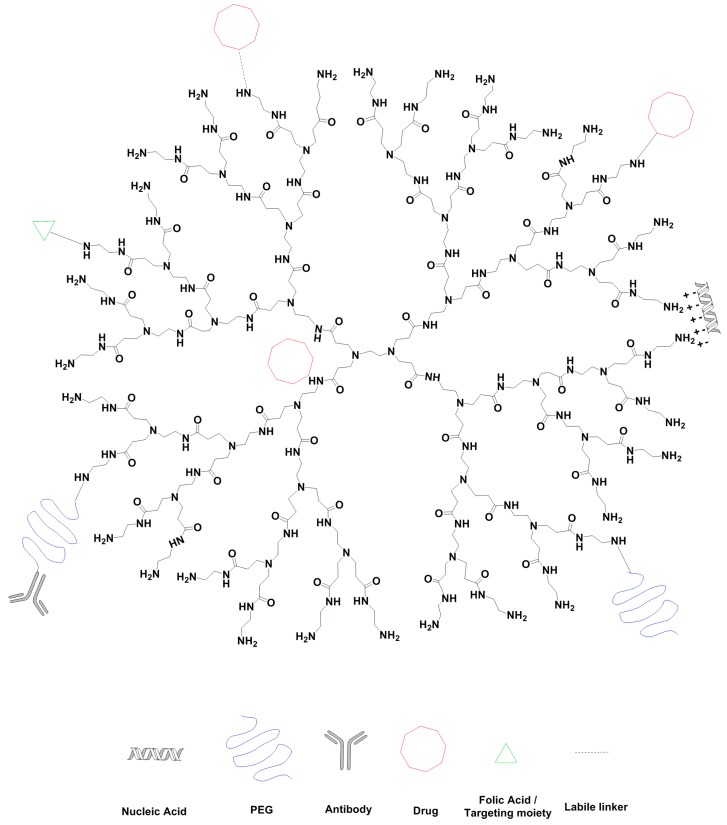
Schematic representation of 3G PAMAM and examples of how dendrimers can interact with drugs and nucleic acid. Also, possible surface modifications that increase tumor specificity. Illustration generated by ChemDraw Professional Version 16.0.1.4.

Surface modification of dendrimers can be useful to improve their safety and can be easily achieved through conjugation of molecules with the reactive terminal groups on these nanocarriers. Polyethylene glycol (PEG) is frequently used to increase plasma circulation time and tumor accumulation through the enhanced permeability and retention (EPR) effect [39,40,41]. Linking or conjugation of dendrimers with PEG chains has been shown as an important step in reducing the cytotoxicity of dendrimers [42,43,44,45]. Jevprasesphant et al. investigated the influence of surface modification by lauroyl chains and PEG_2000_ chains in various generations of PAMAM dendrimer. A remarkable decrease in cytotoxicity towards Caco-2 cells was observed with surface modified dendrimers [43]. Bhadra et al. also found that pegylation on G4 PAMAM dendrimers significantly decreased the hematological toxicity of non-modified PAMAM dendrimers. The pegylation of dendrimers did not impede the drug loading ability. On the contrary, it reduced the leakage of drug from formulation [42]. Besides, shielding of cationic charges by acetylation and hydroxylation could also decrease the toxicity [46,47]. Other modifications can impact cancer targeting properties, for instance, following conjugation with tumor specific antibodies [48,49] and folic acid [50,51] (Figure 2). Besides providing advantages related to the tumor specificity, these alterations usually decrease the number of reactive groups on the surface of dendrimers and, consequently, their charge, thus reducing their toxicity related to their cationic features.

Many kinds of dendrimers have been explored for nucleic acid or drug delivery, including poly(amidoamine) dendrimers [52,53,54,55,56,57], poly(propylene imine) dendrimers [58,59,60], poly(l-lysine) dendrimers [61,62,63], carbosilane dendrimers [64,65,66], and triazine dendrimers [67,68,69]. In this review, we focused on dendrimers that are more commonly used in research, such as PAMAM, PPI, and PLL dendrimers (Table 1, Figure 1).

## 2. Dendrimers for Nucleic Acid Delivery in Cancer Therapy

Nucleic acid therapeutics have already aroused considerable attention because of their biocompatibility and specificity compared to generic chemotherapies. However, nucleic acids are large hydrophilic molecules, which cannot penetrate through cell membranes and are vulnerable to enzymatic degradation in the blood stream. Thus, delivery systems that protect nucleic acid molecules and deliver them to desired locations are necessary for successful nucleic acid therapies. Usually, two types of carriers are used for nucleic acid delivery: viral and non-viral based vectors. However, with greater transfection efficiency, viral vectors generate concerns of immunological and oncological adverse effects that prevent clinical applications. In contrast, non-viral vectors are composed of natural or synthetic molecules that cause low immune responses. These vectors are easy to fabricate and can be modified with various targeting moieties towards diverse organs. Such advantages make non-viral vectors ideal platforms for nucleic acid delivery. 

**Figure 3:**
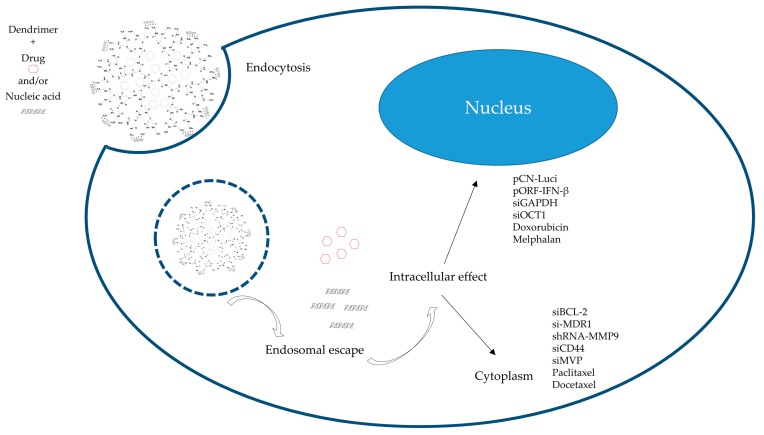
Schematic representation of a PAMAM dendrimer as drug and/or nucleic acid delivery platforms. Dendrimers are internalized by the cell into endosomal vesicles. Due to the lower pH inside the endosome (pH 5.5) and the abundant number of tertiary amines in the dendrimers, the “proton sponge effect” is achieved. It promotes the influx of ions into the vesicles, which eventually leads to rupture of the endosomal wall and release of the cargo intracellularly. Depending on the payload, the effect will be exerted in the cytoplasm or in the nucleus.

Among those non-viral vectors, dendrimer-based vectors have drawn great interest for over two decades as potential nucleic acid delivery systems. Several distinctive properties of dendrimers make them preferable to other cationic polymers. First, a high density of cationic charge provides multiple attaching sites for nucleic acid molecules. Chen et al. postulated that the high density of cationic charges in dendrimers played an important role in assisting DNA molecule complexation [70]. Second, protection of nucleic acid from nuclease is one of the major concerns in nucleic acid delivery. Various groups have proved that complexation of nucleic acid molecules with dendrimers protected nucleic acid molecules from enzymatic degradation [71,72,73]. Third, an abundant amount of tertiary amines in dendrimer structures facilitates the endosomal escape of nucleic acid molecules through a “proton sponge” effect [74,75]. It is believed that tertiary amines would get protonated inside of endosomes, into which negatively charged ions, such as chlorine, fluxed. This effect disrupts the endosome membrane, facilitating the release of cargo molecules (Figure 3). Last but not least, the surface of dendrimers is suitable for modification with functional groups as well as targeting moiety. Potential ability in the delivery of nucleic acid to target location makes dendrimers superior to traditional linear polymers, regarding biological and chemical properties.

Table 2 presents a summary of the dendrimer-based nanosystems described further in this section.

### 2.1. PAMAM Dendrimers for Nucleic Acid Delivery

In past two decades, PAMAM dendrimers have received the most interest among all dendrimers [29]. Haensler and Szoka first reported PAMAM dendrimers used in nucleic acid delivery in 1993. They found that PAMAM complexed with DNA plasmid by electrostatic interaction. The dendriplexes formed possessed a better cell internalization ability and released DNA cargo by taking advantage of a “proton sponge” buffering effect [84]. After that, different generations of PAMAM were tested for their ability in the delivery of nucleic acid molecules. 

The transfection efficiency of PAMAM is affected largely by the generation of the dendrimer, which determines the structure of the PAMAM molecule. In summary, low generation PAMAM has fewer surface primary amines and looser surface structure, while high generation PAMAM has much more surface primary amines that form a surface with high density [85]. For example, G1 PAMAM is not able to complex nucleic acid because of the low positive charge density. The structure of G2 PAMAM tends to be more planar-elliptical [86,87]. G2 PAMAM is able to complex with DNA, but the transfection efficiency is lower than G3–G5 [70]. However, G2 PAMAM has been combined with another delivery platform to optimize its therapeutic efficiency. AM Chen et al. applied G2 PAMAM onto the surface of mesoporous silica nanoparticles and used this system in the delivery of Bcl-2 siRNA in A2780/ADR cells [88]. 

Structures of PAMAM with higher generations become more compact and spherical. Such construction provides a surface with a high density of primary amines in binding with nucleic acid molecules. For example, Perez et al. reported successful downregulation of green fluorescent protein (GFP) in both T98G-EGFP and J774-EGFP cells by using siGFP complexed with PAMAM G7 [57]. This group also showed that this PAMAM G7/siRNA system could enhance the delivery into the brain by intranasal delivery with in situ-forming mucoadhesive gels [89]. Several other groups also studied the structural polymorphism as well as the transfection efficiency of PAMAM/nucleic acid complexes [86,90,91].

Overall, G3-G10 PAMAM dendrimers form more stable dendriplexes with nucleic acid molecules. Also, the transfection efficiency increases with the increment in generations [55]. However, with a more rigid structure and more primary amines exposed on the molecule surface, PAMAM with high generations results in higher toxicity, including erythrocyte lysis [92]. Such toxicity is related with the primary amines rather than secondary or tertiary amines. In order to minimize the toxicity, various modifications have been applied to the surface [43,93].

The balance between positive charges and nucleic acids affinity determines the therapeutic efficiency and toxicity of PAMAM dendrimers. Among all generations of PAMAM, G3 to G6 are the most widely used. It was believed that toxicity of PAMAM is mainly related to the positively charged primary amines on the surface. These cationic charges interact with the cell membrane and result in membrane disruption via nanohole formation, membrane thinning, and erosion [94]. Shielding or conjugating the primary amines were regarded as central tools in reducing cytotoxicity as well as enhancing the therapeutic performance, even though the variation of PAMAM cores also played a role in altering the molecular structure that would result in toxicity [95]. Zhang et al. synthesized PAMAM with trimesyl cores at different generations and compared their gene transfection efficiency as well as cytotoxicity. Though cytotoxicity of such PAMAM was more severe than PEI or PLL at the same concentration, the author thought that with some modification there was still a possibility of applying them as a gene delivery platform [96]. 

Internally quaternized hydroxyl-terminated PAMAM (QPAMAM-OH) and surface-acetylated PAMAM (QPAMAM-NHAc) have no positively charged group on the surface. However, they have cationic charges inside, which can complex with nucleic acid molecules. Patil et al. synthesized QPAMAM-OH and QPAMAM-NHAc in order to develop a carrier that is less cytotoxic than PAMAM-NH_2_. The quaternized PAMAM-NHAc was reported to have less cytotoxicity but had enhanced intracellular accumulation than PAMAM-NH_2_ [76]. Later, Patil et al. conjugated luteinizing hormone-releasing hormone (LHRH) as a cancer targeting moiety to deliver siBCL-2 to show efficient cancer cell penetration and low cytotoxicity. However, only the targeted vehicle successfully downregulated BCL-2 expression by around 80% [77]. This may explain the importance of tertiary amines in helping the endosomal escape of nucleic acid molecules. One of the advantages of primary amines on a PAMAM is that it can be easily conjugated with different targeting or shielding moieties. These conjugations not only shield the positive charge but also enhance the specific accumulation of nucleic acid molecules inside cells. Different moieties, including alkyl chains with different length [97,98], hyaluronic acid [99,100], Tat peptide [101], α-cyclodextrin (α-CDE) [102,103,104], PEG chains [51,105], arginine [33,80,106], and arginyl glycyl aspartic acid (RGD) targeting peptide [107,108] have been conjugated with different generations of PAMAM. All these modifications may decrease the cytotoxicity and enhance the transfection efficiency to a different extent.

Recently, Chahal and Khan et al. prepared novel dendrimer-RNA nanoparticles for delivery of replicon mRNA, which could self-replicate and generate protective immunity against specific viruses. They synthesized new dendrimer by reacting PAMAM G1 dendrimer with 2-tridecyl-oxirane. This alkylated dendrimer, together with lipid-anchored PEG, formed nanoparticles encapsulating mRNA inside. Successful delivery of replicon mRNA by this system resulted in antibody production and antigen-specific CD8+ T-cell responses against the encoded antigen, providing immunity and survival to lethal pathogen challenge in vivo [78]. Another study from this group also showed that alkylated PAMAM G1 dendrimer could be used in siRNA delivery. Such modified PAMAM dendrimer had a high avidity for Tier2-positive endothelial cells in the lung, serving as a promising platform for nucleic acid delivery to the lung [109].

Besides surface modifications on PAMAM, tri-block modification has also been applied to PAMAM to increase its applicability. Tri-block modifications combine different blocks with different properties together, utilizing their advantages, while minimizing some of their pitfalls. Patil et al. developed a multifunctional triblock nanocarrier consisted of PAMAM G4, PEG (3000 Da), and PLL to deliver siBCL-2. After conjugation with PEG, all primary amines on PAMAM were acetylated. They used the tertiary amines on PAMAM for a “proton sponge” effect rather than complexing with siRNA. A PEG moiety was used to confer nuclease stability in the blood stream, and a PLL moiety was used for complexing with siRNA. This triblock nanocarrier-siRNA complex showed an excellent siRNA protection ability and decreased BCL-2 gene expression by 50% compared to either PLL-siRNA complex or PAMAM-siRNA complex [79]. Biswas et al. conjugated PAMAM G4 with PEG (2000 Da), which was linked with 1,2-Dioleoyl-sn-glycero-3-phosphoethanolamine (DOPE). PAMAM served as a siRNA complexing moiety and DOPE was used to create hydrophobic cores where small hydrophobic molecules, such as doxorubicin (DOX), were encapsulated [110]. Elsabahy et al. developed a polyion complex micelle to deliver siRNA. This complex micelle was comprised of a PAMAM/siRNA core, to which a detachable triblock polymer (poly(propyl methacrylate-co-methacrylic acid)-PEG-monoclonal antibody) was electrostatically linked. Under an acidic endosomal environment, this complex micelle had the polymeric shell detached and PAMAM/siRNA cargo released. Experimental results showed that this multiblock polymer was efficiently internalized and significantly decreased BCL-2 expression in PC-3 cells [111,112].

### 2.2. PPI-Based Dendrimers for Nucleic Acid Delivery

PPI dendrimers also have abundant primary amines that assist complexing nucleic acid molecules on their surfaces. However, not much research has been done on PPI nucleic acid delivery systems compared to PAMAM dendrimers. Zinselmeyer et al. established that low generations of PPI were efficient DNA delivery carriers in vitro [113]. Besides, Kabanov et al. demonstrated that the DNA binding affinity with PPI dendrimers are generation dependent [114]. Overall, PPI G2 had the lowest cytotoxicity and highest transfection efficiency in DNA delivery [113]. For delivery of smaller nucleic acid molecule, such as RNA delivery, PPI G4 is preferable. Taratula et al. showed that PPI dendrimers of higher generations like G4 and G5 effectively initiated the complexation of siRNA, resulting in enhanced downregulation of target mRNA in A549 cells [115]. However, the application of PPI dendrimers is still restricted by its toxicity because of their exposed cationic charges.

In order to decrease the cytotoxicity, PPI dendrimers were either quaternized or modified to shield the cationic charges. Schatzlein et al. reacted the primary amines on PPI with methyl iodide and used this quaternized PPI for DNA delivery. They reported that quaternized PPI G2 dendrimers were very well tolerated and efficiently delivered the gene to liver tissue in vivo [116]. Taratula et al. crosslinked a PPI/siRNA complex with dithiol-containing molecules and coated with LHRH peptide conjugated PEG chains. This PEG chain modification improved the stability and intracellular bioavailability in plasma. Meanwhile, LHRH peptide enhanced the uptake and accumulation of complex inside of cancer cells, leading to an efficient gene silencing effect [60]. 

Another strategy for improving the transfection performance of PPI dendrimers is to combine them with nanoparticles. Chen et al. physically attached PPI G3 dendrimers to gold nanoparticles, which served as a discrete core for siRNA complexation. In the final formulation, PPI was complexed with siRNA while gold nanoparticles were separated from the complexes. This approach showed promising efficiency in siRNA-mediated mRNA downregulation [81]. Taratula et al. also used supermagnetic iron oxide nanoparticles, together with the incorporation of LHRH conjugated PEG chains for complexation between PPI G5 and siRNA. Such modification improved the serum stability and selective internalization of siRNA therapeutics, resulting in an increased targeted gene silencing effect [117].

### 2.3. Poly Amino Acid Based Dendrimers for Nucleic Acid Delivery

Given that the primary amines of amino acids, dendritic poly amino acids can also be used as gene delivery vectors. For example, lysine plays an important role in wrapping DNA around histones inside the nucleus. PLL dendrimers have been developed as non-viral vectors for siRNA delivery. Osaki et al. studied the transfection efficiencies of PLL from generations 1 to 7. They reported that PLL G6 is the most favorable transfection agent [32]. PLL dendrimers of low generations have fewer primary amines, which was not optimal for complexing large nucleic acid molecules. Compared to linear PLLs with the same amount of lysine residues, dendritic PLLs have a much higher transfection efficiency, even though linear PLLs condense DNA molecules better. The reason is that PLL dendrimers could be regarded as “proton sponge effect” initiative materials, which facilitate the endosomal escape of the DNA/PLL complex. Also, the weaker interaction between DNA molecules and PLL dendrimers makes DNA molecules more accessible to RNA polymerase and easier to be expressed [118].

Despite the fact that PLL dendrimers have been reported to be non-immunogenic and biocompatible [119], excessive positive charges on PLL dendrimers can still bind to serum proteins as well as red blood cells. Thus, steric stabilization using PEG was applied to avoid these situations. Different groups have conjugated a PEG block on PLL dendrimers, showing that an appropriate portion of PEG stabilized the dendrimer/DNA complex structures without affecting the DNA complexing ability [61,82]. 

Other modifications were applied to PLL for improved transfection efficiency. Inoue et al. combined a weak-base amphiphilic peptide, EndoPorter, with PLL G6 dendrimer. They used this system to deliver different types of siRNA, and effectively knocked down GAPDH, PEPCK (a rate-limiting enzyme for gluconeogenesis), and organic cation transporter 1 (OCT1), reducing the glucose production of rat hepatoma H4IIEC3 cells [62].

Okuda et al. substituted lysine residues on the outer surface of PLL G6 for another positively charged amino acid, such as histidine or arginine. Replacement of lysine with arginine increased the transfection efficiency by 3- to 12-fold in several cell lines, while retaining the plasmid DNA binding ability. However, PLL dendrimer with histidine replaced lost its transfection ability [83]. A similar strategy of decorating the surface of dendrimers with arginine residue was widely used in PAMAM and PPI dendrimers [33,58,80,106]. Arginine conjugated dendrimer has shown enhanced transfection efficiency and low toxicity.

## 3. Dendrimers as Drug Delivery Carriers in Cancer Therapy

The ease of control of the properties of dendrimers including structure and size, aqueous solubility, monodispersity, and the various options of terminal functional groups render them with high drug delivery ability [120]. Drugs can be carried by dendrimers using different strategies, which can be separated basically into chemical and physical interactions (Figure 2) [121]. Physical interactions rely on entrapment of the drug in the dendrimer core by noncovalent association through hydrogen bonds, hydrophobic, or electrostatic interactions. The internal cavities of dendrimers, a characteristic of their structure, are in most cases hydrophobic and allow the interaction with poorly soluble drugs. Sanyakamdhorn et al. described a thorough investigation of the intramolecular interaction of DOX and tamoxifen, and their respective analogs, with dendrimers [122,123]. They showed that the formation of hydrogen bonds between the drugs and the -NH groups in the interior of PAMAM, as well as hydrogen bonding and electrostatic interactions with surface amino groups, were responsible for the physical encapsulation of the drugs. This type of association, however, is limited by a rapid release that can occur before the carriers reach their target [124,125]. Chemical interactions, on the other hand, involve the covalent conjugation of the drugs with the functional terminal groups of the dendrimers and appear to be more stable [125,126]. “Smart” strategies use labile linkages that will be cleaved upon exposure to a specific environment and release the drug at the target site [34,127]. Various potent anticancer drugs—including paclitaxel (PTX), camptothecin, methotrexate, 5-fluorouracil, and DOX free base—are known for their high hydrophobicity, which leads to difficulties in finding an adequate vehicle for their administration. In addition, these drugs are associated with toxic effects due to lack of tumor specificity. Dendrimers have emerged in as promising platforms for these molecules [41,128,129,130,131] and Table 3 presents a summary of the main studies discussed in this section.

### 3.1. PAMAM Dendrimers for Drug Delivery

DOX is one of the most widely used antitumor drugs. Despite showing good efficacy, it is also well known for its systemic side effects, mainly cardiomyopathy [136,137]. Various groups have worked on the development of dendrimer-based nanosystems to optimize the therapy, thus increasing efficacy and reducing the toxic effects of DOX [45,138,139,140]. To improve the drug accumulation in lung tumors, Zhong et al. [131] prepared DOX-dendrimer conjugates and investigated their ability to decrease metastatic lung burden through local administration of this nanosystem. PAMAM of generation 4 was used, and DOX was conjugated to their surface through acid-sensitive hydrazone bonds. The pulmonary administration of DOX-dendrimer conjugate in mouse-bearing melanoma B16-F10 cells as a model of lung metastasis promoted decreased tumor burden, enhanced accumulation in the lungs, and diminished the distribution to the cardiac tissue. The use of acid-sensitive hydrazone bonds to conjugate DOX is an approach to develop stimuli-sensitive carriers that will release their cargo when exposed to low pH, such as in the tumor microenvironment or endosomal vesicles [141], thus improving tumor specificity.

Other stimuli-sensitive approaches can be used to explore the tumor microenvironment characteristics with an aim to increase the specificity of the drug delivery systems [142,143]. Using one of these strategies, Satsangi et al. [132] developed PAMAM dendrimers of fourth generation conjugating PTX through a glycine-phenylalanine-leucine-glycine peptide linker that is cleavable by cathepsin B, an enzyme upregulated in metastatic breast cancer. This PTX-dendrimer conjugate (PGD) showed higher cytotoxicity than free PTX in cells that had greater cathepsin B activity. In normal kidney cells (BUMPT), however, with low cathepsin B presence, PGD did not present cytotoxicity. These in vitro cytotoxicity results were translated in an in vivo tumor inhibition growth assay. PGD had superior efficacy to free PTX to prevent tumor growth in mice implanted with MDA-MB-231 (high expression of cathepsin B) xenografts [132].

The surface of PAMAM dendrimers can be easily modified with various molecules to improve cancer therapy. Active targeting is a strategy that has been used for years to improve tumor specificity and decrease systemic toxicity [144]. The use of antibodies as targeting molecules is one of these approaches. Kulhari et al. [48] developed G4 dendrimers, encapsulating docetaxel (DTX) modified with trastuzumab (TZ) on their surface using PEG as a linker. TZ is used as immunotherapy in tumors that express high levels of the human epidermal growth factor receptor type 2 (HER2) to block downstream signaling [145]. In their work, Kulhari and co-workers compared the in vitro efficacy of TZ-DTX-dendrimers with DTX-dendrimers and free DTX in MDA-MB-453 (HER2-positive) and MDA-MB-231(HER2-negative) cells. The uptake of TZ-DTX-dendrimer was 70% higher than that DTX-dendrimer after 4 h of incubation in the HER-positive cells, whereas no difference was observed between them in HER-negative ones. Cytotoxicity of modified dendrimer was also higher in MDA-MB-453. The IC_50_ of TZ-DTX-dendrimer was 3.6-times higher than DTX-dendrimer, and no difference was observed between any of them or free drug in MDA-MB-123 [48].

In general, it can be observed from the literature that higher generations (4 and up) of PAMAM show better efficacy, since it allows higher drug loading, either by physical or chemical interactions. Higher generations provide more space within the dendritic cavities for the interaction of guest molecules with the tertiary amines than lower ones [21,146]. The same can be assumed for chemical conjugation since the number of modifiable surface groups that are used to conjugate the drugs is increased in high generations [21]. It is, however, important to balance the efficacy of higher generations with their toxicity and considering this, many studies focus on 4G PAMAM as drug carriers, as we can see from the aforementioned research. 

### 3.2. PPI Dendrimers for Drug Delivery

Kesharwani and co-workers investigated the encapsulation of melphalan in PPI dendrimers of different generations and after surface modification [133,147]. Melphalan is a chemotherapy agent that causes DNA alkylation, leading to inhibition of the synthesis of DNA and RNA. When encapsulated in PPI of generations 3, 4, and 5, it showed improved tumor growth inhibition and increased survival rates in BALB/c mice inoculated with MCF-7 cells, especially for G4 and G5. However, the higher the generation, the higher the hemolytic toxicity of these dendrimers was, highlighting the limitations of the use of these carriers due to their cationic nature [147]. When dendrimers were surface-modified with folic acid to increase cancer targeting ability, the biocompatibility increased, probably due to shielding of some cationic groups by folate. Still, lower biocompatibility was seen for G5 compared to G3 and G4. Also, folate-modified dendrimers showed even better tumor growth inhibition in MCF-7 tumor-bearing BALB/c mice [133].

In an attempt to increase tumor specificity, and consequently treatment efficacy, Jain and co-workers [49] developed G4.5 PPI dendrimers that were carboxylic acid terminated to conjugate the monoclonal antibody mAbK1 and to encapsulate PTX (mAbK1-PPI-PTX). mAbK1 targets mesothelin protein, overexpressed in some cancers, but not in normal cells. Their in vitro evaluation in the OVCAR-3 ovarian cell line (which expresses high amount of mesothelin) showed higher cytotoxicity when immunodendrimers were used compared to free drug and PPI-PTX dendrimers. In a xenograft mouse model with BALB/c mice bearing OVCAR-3 cells, the mAbK1-PPI-PTX formulation promoted higher tumor growth inhibition, as well as survival rates, when compared to control, free PTX, and PPI-PTX only. Biodistribution studies showed at least a 7-times higher accumulation of both targeted and non-targeted formulations in the tumor than free PTX during all time points (1, 4, 8, and 24 h post injection). Both in vitro and in vivo evaluation of toxicity of dendrimers vs. immunodendrimers showed that the second approach improved biocompatibility. These results indicate that the use of targeted dendrimers not only improved their efficacy but also decreased their intrinsic toxic profile. 

### 3.3. PLL Dendrimers for Drug Delivery

PLL dendrimers, despite being less well studied than PAMAM or PPI, also represent good potential in the drug delivery field. In the scope of cancer therapy, they are found to be greatly associated with DOX and improve the drug’s anticancer activity, while showing fewer adverse events than the free drug [34,40,134]. As one example of this studies, Al-Jamal and co-workers investigated the potential of these dendrimers as DOX carriers [134]. This group had previously found that cationic PLL dendrimers of generation 6 had good antiangiogenic activity in an in vivo B16F10 xenograft model even in the absence of a therapeutic molecule [148]. By associating the PLL dendrimers with DOX, they attempted to combine the anti-cancer effect of the drug and antiangiogenic effect of the carrier [134]. They found that these dendrimers were able to complex with DOX (DM-DOX) and showed deeper penetration in 3D tumor spheroid models of DU145 prostate cancer cells compared to the free drug. In a B16F10 melanoma xenograft mouse model, they showed that DM-DOX significantly suppressed tumor growth and increased survival compared to free DOX after a single intravenous injection. Penetration of dendrimers in deeper layers of 3D spheroid models and tumors has been attributed to their small size (usually below 10 nm) and positive charge [149,150,151]. Li and co-workers interestingly showed that a size transition from 80 nm pH-sensitive nanoparticles comprised of dendrimer building blocks improved tumor penetration in vitro (spheroids) and in vivo after they were dissociated into 10 nm dendrimers in a slightly acidic environment [150]. 

In another study utilizing sixth generation PLL dendrimers carrying DOX, Niidome et al. [135] found better tumor accumulation of the dendrimers and enhanced tumor inhibition with no apparent toxicity in a mouse model of BALB/cN mice implanted with Colon-26 mouse rectum carcinoma cells. These dendrimers were modified with PEG-linked hydrophobic pentapeptides. PEGylation improved tumor accumulation through the EPR effect, while the oligopeptide link created a hydrophobic cavity to increase DOX encapsulation.

In a more advanced research stage, a formulation of PEGylated PLL dendrimer containing docetaxel conjugated to their surface is currently in Phase I clinical trials. The formulation developed by Starpharma (Melbourne, Australia), named DEP^®^ docetaxel, presented improved tumor targeting and effectiveness against different solid tumors—including breast, ovarian, prostate, and lung—compared to Taxotere, the docetaxel formulation used in the conventional chemotherapy [152]. These results reflect positively on the great expectations for the potential of dendrimers to be used in nanotechnology-based cancer therapy.

## 4. Co-Delivery of Drugs and Nucleic Acid by Dendrimers

Combination therapy is currently one of the most promising approaches in cancer treatment. The association of an anticancer drug with oligonucleotides targeting resistance mechanisms or other targets related to invasion and progression hold the advantages of the enhanced effect that can be obtained from the synergism of both payloads. Many studies have successfully explored this strategy and have shown that the combined therapeutic response is higher than with the single treatment [153,154,155,156]. As shown in the sections above, dendrimers have been extensively used as carriers for drugs or siRNA and have the potential to combine both payloads in one formulation. However, not many studies have reported on the combination of anticancer agents and siRNA in dendrimer-based systems. The studies discussed in this section are summarized and presented in Table 4.

It is known that PAMAM with amino-terminal groups can be easily decorated with molecules that improve their safety, stability, transfection efficacy, and drug loading capability [106,161]. Biswas and co-workers modified G4 PAMAM with poly(ethylene glycol)-dioleoylphosphatidyl ethanolamine (PEG_2000_-DOPE) and mixed it with PEG_5000_-PE to form a micellar-dendrimer system. The presence of the phospholipid contributed to a higher drug loading of DOX in the hydrophobic core of this micelle-like system, while the cationic surface of PAMAM allowed condensation with siRNA. As an initial proof-of-concept work, the authors showed that this delivery system had good serum stability, besides high cellular uptake of drug and siRNA, which led to a better protein downregulation when compared to non-modified dendrimers [110]. This work presents an encouraging platform as a nanocarrier for co-delivery of anticancer drugs with a therapeutic siRNA.

Another study used PAMAM encapsulating DOX but now associated with siRNA targeting major vault protein (MVP). MVP overexpression in cancer cells is involved in transporting drugs away from the nucleus, and is thus related to multidrug resistance. A fifth generation PAMAM was decorated with hyaluronic acid (HA) to increase tumor cell specificity since receptors for HA are overexpressed in many cancer cells. In vitro assays showed that there was an increase in cytotoxicity in MCF7/ADR cells when DOX PAMAM-HA was used in combination with MVP-siRNA, indicating the chemosensitization from the downregulation of MVP protein and an enhanced effect of DOX when associated in the dendrimer system. In vivo pharmacokinetics showed that DOX PAMAM-HA prolonged the concentration of the drug in the plasma of animals and increased its tumor accumulation compared to the free drug [99].

In an attempt to address other drawbacks of conventional chemotherapy and obtain a platform for combination targeting therapy, Shah et al. developed a nanodevice based on PEGylated PPI dendrimers conjugating PTX and complexing siRNA that target CD44 mRNA. This system was also modified with LHRH peptide to promote tumor targeting. Mice were implanted with cancer cells derived from ascitic fluid of patients with metastatic ovarian cancer. The treatment of these animals with these multifunctional nanosystems caused almost complete inhibition of tumor growth [160]. The possibility of an association of siRNA, drug, and LHRH allowed by the versatility of the PPI dendrimers was crucial for this effect.

In a different approach, Wang and collaborators used branched PEG (bPEG) conjugated with G2 PAMAM (PSPG) through disulfide linkages (cleavable by intracellular glutathione) to encapsulate DOX and siRNA against B-cell lymphoma 2 (Bcl-2) [157]. The bPEG promoted a shielding effect, which protected the siRNA against degradation compared to G2 PAMAM only. In vivo experiments demonstrated a superior effectiveness of the combinatorial approach PSPG/DOX/Bcl-2 when compared to PSPG/DOX or PSPG/Bcl-2 alone, with no increase in tumor volume after 22 days of treatment and no side effects. In a later study, this research group combined PTX and siRNA against TR3/Nur77, an orphan nuclear receptor and new therapeutic target for pancreatic cancer therapy [158]. They targeted this dendrimer-based nanosystem with plectin-1 peptide (PTP), a biomarker for pancreatic cancer, and showed that the targeted system increased tumor accumulation and retention time after intravenous injection. Additionally, there was a substantial improvement in tumor inhibition when compared to the non-targeted co-delivery system, highlighting the specificity promoted by the targeting strategy.

Gu et al. reported a co-delivery system of DOX and short hairpin RNA (shRNA) targeting MMP-9 enzyme comprised of graphene oxide (GO) functionalized with G3 PAMAM to ally the high drug loading capacity of GO with the efficient transfection ability of the polymer [159]. The authors showed in vitro synergism of the co-delivery approach in MCF-7 breast cancer cells and increased biocompatibility in vivo compared to linear polyethyleneimine, a commonly used gene transfection agent.

## 5. Conclusions and Future Perspectives

Dendrimers are potential carriers that can work as a platform for drug solubilization and nucleic acid condensation, allowing enhanced efficacy in cancer therapy and/or reduced toxicity compared to the conventional chemotherapeutic treatments. Their structural properties and the fact that they can be almost precisely controllable during their synthesis are facts that support their application in the delivery field for cancer. Regarding the safety of these nanosystems, it is clear that their hemolytic and toxic effects must be addressed and many studies approach those drawbacks by modifying the architecture of the dendrimers, especially their surface. In many cases, the surface modifications are intended to promote long circulation or to improve cancer targeting. While obtaining a system with higher specificity, they ultimately generate safer nanoparticles since these alterations shield the cationic groups associated with toxicity. The use of dendrimer-based systems for antitumor therapy is advantageous, and much remains to be explored and addressed to validate their clinical use with the aim of improvement in the efficacy of treatment and safety for patients. Despite the approval of some nanoparticle-based formulations for cancer treatment and the increase in the number of clinical trials involving these types of delivery systems, the clinical translation of dendrimers has not yet been achieved. As mentioned in the review, only one anti-cancer formulation has advanced from preclinical studies to Phase I clinical trials, and that is a reflex of the hurdles encountered in the bench-to-bedside transition. DEP^®^ docetaxel has shown good progress and promising results, and the success in its clinical trials could encourage more investment to take other dendrimer platforms to next phases. The optimization of all processes in dendrimer-based formulations production is necessary for that, from the synthesis of the polymers, the reproducibility regarding drug/gene loading and stability, to the safety and efficacy of the carriers. Furthermore, superior therapeutic response over the existing treatments is expected to justify the advancement of such nanoformulations. Dendrimers show a great potential, especially in the promising field of RNAi, which has not yet reached the market either. Many advances have been made to obtain safe and efficacious dendrimer-based formulation, but it is imperative to continue the optimization of these versatile platforms to meet medical needs and market standards.

## Figures and Tables

**Table 1:** Physical characteristics of PAMAM and PPI dendrimers based on ideal defect-free structures

Generation	Molecular Weight (g/mol)		Size (nm)		Number of Surface Groups	
	PAMAM	PPI	PAMAM	PPI	PAMAM	PPI
1	1430	317	1.9	0.9	8	4
2	3256	773	2.6	1.4	16	8
3	6909	1687	3.6	1.9	32	16
4	14,215	3514	4.4	2.4	64	32
5	28,826	7168	5.7	2.8	128	64

**Table 2:** Examples of dendrimer-based nanosystems as vehicles for nucleic acid

Polymer	Generation	Payload	Application	Modification	Reference
PAMAM	G4	siBCL-2	Ovarian cancer	Hydroxylated LHRH peptide	[76,77]
	G1	Replicon mRNA	Vaccine	2-tridecyloxirane	[78]
	G4	siBCL-2	Ovarian cancer	PEG-PLL	[79]
	G4	IFN-β	Malignant glioma	Arginine	[80]
PPI	G5	siBCL-2	LHRH positive cancer	DTBP LHRH-PEG	[60]
	G2	pCN-Luci		Arginine	[58]
	G3	siBCL-2	Lung cancer	Au nanoparticle	[81]
PLL	G3-G6	p-Luci		PEG	[82]
	G6	siGAPDH siOCT1	Gluconeogenesis	EndoPorter	[62]
	G6	pCMV-Luc		Arginine	[83]

**Table 3:** Examples of dendrimer-based nanosystems as drug carriers

Polymer	Generation	Payload	Application	Modification	Reference
PAMAM	G4	Doxorubicin	Lung metastasis	-	[131]
	G4	Paclitaxel	Breast cancer	-	[132]
	G4	Docetaxel	Breast cancer	Trastuzumab	[48]
PPI	G3–G5	Melphalan	Breast cancer	Folic acid	[133]
	G4.5	Paclitaxel	Ovarian cancer	mAbK1	[49]
PLL	G6	Doxorubicin	Melanoma	-	[134]
	G6	Doxorubicin	Rectum cancer	Pentapeptide-PEG	[135]

**Table 4:** Examples of dendrimer-based nanosystems as carriers of drug and nucleic acids simultaneous.

Polymer	Generation	Payload	Application	Modification	Reference
PAMAM	G2	Doxorubicin + siBCL-2	B-cell lymphoma	PEG	[157]
	G2	Paclitaxel + siTR3	Pancreatic cancer	Plectin-1 peptide	[158]
	G3	Doxorubicin + shMMP-9	Breast cancer	Graphene oxide	[159]
	G4	Doxorubicin + siGFP	-	-	[110]
	G5	Doxorubicin + siMVP	Breast cancer	Hyaluronic acid	[99]
PPI	G5	Paclitaxel + siCD44	Ovarian cancer	Luteinizing hormone-releasing hormone-PEG	[160]
